# Lung Matrix Metalloproteinase Activation following Partial Hepatic Ischemia/Reperfusion Injury in Rats

**DOI:** 10.1155/2014/867548

**Published:** 2014-01-23

**Authors:** Giuseppina Palladini, Andrea Ferrigno, Vittoria Rizzo, Eleonora Tarantola, Vittorio Bertone, Isabel Freitas, Stefano Perlini, Plinio Richelmi, Mariapia Vairetti

**Affiliations:** ^1^Department of Internal Medicine and Therapeutics, Fondazione IRCCS Policlinico S. Matteo, University of Pavia, Via Ferrata 9A, 27100 Pavia, Italy; ^2^Department of Molecular Medicine, Fondazione IRCCS Policlinico S. Matteo, University of Pavia, 27100 Pavia, Italy; ^3^Department of Biology and Biotechnology “Lazzaro Spallanzani,” University of Pavia, 27100 Pavia, Italy; ^4^Institute of Molecular Genetics of the C.N.R. (IGM-CNR), Histochemistry and Cytometry Section, University of Pavia, 27100 Pavia, Italy

## Abstract

*Purpose*. Warm hepatic ischemia-reperfusion (I/R) injury can lead to multiorgan dysfunction. The aim of the present study was to investigate whether acute liver I/R does affect the function and/or structure of remote organs such as lung, kidney, and heart via modulation of extracellular matrix remodelling. *Methods*. Male Sprague-Dawley rats were subjected to 30 min partial hepatic ischemia by clamping the hepatic artery and the portal vein. After a 60 min reperfusion, liver, lung, kidney, and heart biopsies and blood samples were collected. Serum hepatic enzymes, creatinine, urea, Troponin I and TNF-alpha, and tissue matrix metalloproteinases (MMP-2, MMP-9), myeloperoxidase (MPO), malondialdehyde (MDA), and morphology were monitored. *Results*. Serum levels of hepatic enzymes and TNF-alpha were concomitantly increased during hepatic I/R. An increase in hepatic MMP-2 and MMP-9 activities was substantiated by tissue morphology alterations. Notably, acute hepatic I/R affect the lung inasmuch as MMP-9 activity and MPO levels were increased. No difference in MMPs and MPO was observed in kidney and heart. *Conclusions*. Although the underlying mechanism needs further investigation, this is the first study in which the MMP activation in a distant organ is reported; this event is probably TNF-alpha-mediated and the lung appears as the first remote organ to be involved in hepatic I/R injury.

## 1. Introduction

Reperfusion following prolonged ischemia may cause paradoxical damage at several levels. This phenomenon, defined as ischemia-reperfusion (I/R) injury, has been described in the heart as well in other organs, such as the liver [1]. Among the several involved mechanisms, oxygen toxicity and free radical production play an important role [2]. Warm hepatic I/R injury may occur not only during liver transplantation, but also following surgical resections requiring hepatic inflow occlusion for bleeding control. Via systemic yet poorly understood mechanisms, I/R injury in one organ can also lead to multiorgan dysfunction that is to tissue damage in remote organs away from the body district where the I/R damage is taking place. For example, I/R intestine damage has been described to cause multiple organ dysfunction due to uncontrolled production and release of cytokines and other proinflammatory molecules [3]. Recent data suggested that also hepatic I/R injury may cause damage to remote organs such as kidney [4], heart [5], and lung [6]. Indeed, damaged liver tissue releases destructive proinflammatory cytokines and oxygen-derived radicals into the circulation that are likely causing further damage to remote organs [7]. In an experimental model, Colletti et al. [8], showed that hepatic I/R injury is associated with lung dysfunction such as neutrophil infiltration, edema, intraalveolar hemorrhage and endothelial activation.

Inflammatory cytokines such as TNF-alpha participate to extracellular matrix (ECM) degradation following liver injury, and hepatic TNF-alpha expression has been demonstrated to be parallel with induction of matrix metalloproteinases (MMPs) [9], collagenolytic zinc-dependent enzymes that degrade several ECM constituents such as collagen, gelatin, elastin, and fibronectin [10]. A recent study [11] reports increased liver MMP-9 expression following I/R injury, and a correlation between serum MMP-9 and severity/progression of liver damage has been described in the setting of I/R injury [12], acute allograft rejection [13], and chronic viral hepatitis [14]. Also in the kidney, it has been recently shown that MMPs play a role in the development of endothelial damage during ischemia-reperfusion injury, with increased MMP-9 activity paralleling degradation of endothelial cells and the subsequent increase in vascular permeability [15]. Cardiovascular dysfunction frequently occurs after major surgery or liver transplantation. Several investigators have reported that liver ischemia is associated with the release of vasoactive substance that inflicts remote cardiac damage [16].

The mechanisms leading to the initiation of multiorgan injury have not yet been elucidated. The aim of the present study was therefore to investigate whether acute liver ischemia and reperfusion do affect the function and the structure of remote organs such as kidney, heart, and lung via the modulation of ECM remodelling. As indicators of remote and local tissue damage and leukocyte infiltration we used malondialdehyde (MDA), an indicator of lipid peroxidation rate, and myeloperoxidase (MPO), a neutrophil marker.

## 2. Material and Methods

### 2.1. Animals

The use of animals in this experimental study was approved by the National Institute for Research, and the animals were cared for according to its guidelines. Thirty-two Male Sprague-Dawley rats (200–250 g) with free access to water and food were used.

### 2.2. Materials

All reagents were of the highest grade of purity available and were purchased from Sigma Aldrich.

### 2.3. Ischemia-Reperfusion (I/R) Procedure

The effects of I/R were studied in vivo in a partial normothermic hepatic I/R model that has been previously reported [17, 18]. Briefly, the abdomen was opened by a median incision while the rats were anesthetized with pentobarbital (50 mg/kg). Ischemia to the left and median lobe was induced by clamping the portal vein and hepatic artery with microvascular clips for 30 min, and the abdomen was temporary closed with a suture. After 30 min of ischemia, the abdomen was reopened, the clips were removed, the abdomen was closed again, and the liver was reperfused for 60 min. To prevent postsurgical dehydration and hypotension 1 mL of normal saline was injected in the inferior vena cava immediately after the removal of the clips. The duration of the injection was approximately 30 s. During I/R the animals (*n* = 17) were maintained on warm support to prevent heat loss: rectal temperature was maintained at 37 ± 0.1°C. Sham-operated control animals (*n* = 15) were similarly treated as compared with I/R group for all the aspects of the experimental model: rats were maintained under heat support during anaesthesia of equal length of time, injected with 1 cc saline and submitted to similar manipulation of the liver hilus without vascular occlusion. Blood samples were obtained after reperfusion and immediately centrifuged to isolate serum. At the end of the reperfusion period, tissue samples of the liver, ischemic lobes (left), of the kidney (cortex and medulla), of the heart (left ventricle), and of the lung were snap frozen in liquid nitrogen.

### 2.4. Biochemical Assays

Liver and kidney injury was assessed by serum release of alanine transaminase (ALT), aspartate transaminase (AST), lactate dehydrogenase (LDH), creatinine, and urea by an automated Hitachi 747 analyser (Roche/Hitachi, Indianapolis, IN, USA).

Heart injury was assessed by serum evaluation of highly specific marker of myocardial cell damage such as Troponin I (cTN1) by an automated Hitachi 747 analyser (Roche/Hitachi, Indianapolis, IN, USA).

The amounts of released TNF-alpha in serum were quantified by commercial rat TNF-alpha ELISA kit from R&D Systems (Minneapolis, USA) according to the manufacture instructions.

The amounts of malondialdehyde (MDA) formation were quantified by HPLC method using the Chromsystems assay kit (Chromsystems GmbH, München). The assay was performed according to the manufacturer instructions with some modifications: briefly, the derivatized samples were incubated for 60 minutes at 95°C and finally used after centrifugation [18].

Myeloperoxidase (MPO) activity was measured with a fluorimetric detection kit (Cayman Chemical) after an adequate tissue preparation. Briefly liver, lung, kidney, and heart tissues were homogenized (IKA-Ultraturrax T10) in a cooled 0.02 M potassium phosphate buffer (pH 7.4). After addition of another equal volume of cooled 0.02 M potassium phosphate buffer, the homogenate was centrifuged at 4°C for 15 min at 20.000 rpm in order to pellet insoluble cellular debris [19]. Pellets were resuspended in a cooled 0.05 M potassium phosphate buffer (pH 6) containing 0.5% hexa-1,6-bis-decyltrimethylammonium bromide (HTAB) and homogenized. Samples were sonicated for 30 sec and submitted to two cycles of freeze/thaw. Finally samples were centrifuged at 4°C for 15 min at 20.000 rpm and supernatants were immediately frozen at −80°C for later user. One unit of MPO activity was defined as the amount of enzyme that caused the formation of 1 nmol of fluorophore per minute at 25°C.

### 2.5. Tissue Sources for MMPs Analysis

After sacrifice liver, kidney, heart, and lung were quickly excised and placed in cold (4°C) buffer (30 mM Histidine, 250 mM sucrose, and 2 mM EDTA, pH 7.2) to remove blood. Liver and lung were weighed and subsequently cut, frozen in liquid nitrogen and stored at −80°C, until use. Kidney was cleaned of external tissue; the renal cortex and medulla were separated and subsequently frozen in liquid nitrogen and stored at −80°C, until use. Heart was separated in atria, right and left ventricle. The left ventricle tissue samples were then frozen in liquid nitrogen and stored at −80°C, until use.

#### 2.5.1. Hepatic Protein Isolation

Hepatic MMPs were extracted by homogenisation (IKA-Ultraturrax T10) of frozen liver tissue, in an ice-cold extraction buffer (1 : 10 wt/vol) containing 1% Triton X-100, 500 mmol/L Tris-HCl, 200 mmol/L NaCl, and 10 mmol/L CaCl_2_, pH 7.6 [20]. The homogenate was then centrifuged (30 min at 12.000 rpm at 4°C) and the protein concentration of the supernatant was measured with the colorimetric Lowry method [21]. Samples were stored at −20°C before use.

#### 2.5.2. Renal Protein Isolation

Fifty milligrams of cortex and medulla were used to homogenize in a dissociation buffer containing 10 mmol/L cacodylic acid, 0.15 mmol/L NaCl, 1 mmol/L ZnCl_2_, 20 mmol/L CaCl_2_, 1.5 mmol/L NaN_3_, and 0.01% Triton X-100, pH 5.0 [22]. The homogenate was then shaken at 4°C for 24 h and the protein concentration of the supernatant was measured with the colorimetric Lowry method [21]. Samples were stored at −20°C before use.

#### 2.5.3. Cardiac and Pulmonary Protein Isolation

Left ventricle myocardial and lung samples were homogenized in an ice-cold extraction buffer [23] (1 : 10 wt/vol) containing cacodylic acid (10 mmol/L), NaCl (150 mmol/L), ZnCl_2_ (1 mmol/L), CaCl_2_ (20 mmol/L), NaN_3_ (1.5 mmol/L), and Triton X-100 0.01% vol/vol (pH 5). The homogenate was then centrifuged (5 min at 10.000 rpm) and the supernatant protein concentration was measured with the colorimetric Lowry method [21]. Samples were stored at −20°C before use.

### 2.6. MMPs Zymography

In order to detect MMPs activity present in the samples, the homogenate protein content was normalized by a final concentration of 400 *μ*g/mL in sample loading buffer (0.25 M Tris-HCl, 4% sucrose w/v, 10% SDS w/v, and 0.1% bromphenol blue w/v, pH 6.8). After dilution the samples were loaded onto electrophoretic gels (SDS-PAGE) containing 1 mg/mL of gelatin under nonreducing conditions [24, 25], followed by zymography as described previously [26].

The zymograms were analyzed by densitometer (GS 710 Densitometer BIORAD, Hercules, CA, USA) and data were expressed as optical density (OD), reported to 1 mg/mL protein content.

### 2.7. Tissue Morphology

Liver and lung tissues were fixed in 2% paraformaldehyde in 0.1 M phosphate buffer at pH 7.4 for 24 hours, processed routinely and embedded in Paraplast wax. Sections (8 *μ*m thick) were stained with hematoxylin and eosin (H&E). To appraise the severity of hepatic injury, H&E-stained sections were evaluated as follows: Grade 0, minimal or no evidence of injury; Grade 1, mild injury consisting of cytoplasmic vacuolation and focal nuclear pyknosis; Grade 2, moderate-to-severe injury with extensive nuclear pyknosis, cytoplasmic hypereosinophilia, and loss of intercellular borders; Grade 3, severe necrosis with disintegration of hepatic cords, hemorrhage, occasional granulomas, cytoplasmic pallor, and cellular swelling [27].

To appraise the severity of lung injury, the number of granulocytes was evaluated in H&E-stained sections and calculated per microscopic field [28]: the prepared sections were coded and examined by an independent histologist in a single-blind scoring procedure.

Kidney tissue was frozen in liquid nitrogen and stored at −80°C. Sections (8 *μ*m thick) were stained with H&E. Microscopic criteria for tubular damage are tubular brush border loss, cytoplasmic swelling, and cellular debris.

### 2.8. Statistical Analysis

Results are expressed as mean ± error standard as specified. Comparisons between groups were performed by unpaired *t*-test. When data distribution was not normal, according to the Kolmogorov-Smirnov test, Mann-Whitney test was used. All statistical procedures were performed using the MedCalc statistical software package (12.2.1.0 version). Value of *P* < 0.05 was considered significant.

## 3. Results

### 3.1. Liver I/R Injury

As expected, serum levels of AST, ALT, and LDH increased significantly in animals submitted to ischemia (30 min) and reperfusion (60 min) as compared with sham-operated group (Table 1).

### 3.2. Kidney Function

Serum creatinine and urea did not significantly differ between sham and I/R groups (Table 1).

### 3.3. Heart Biomarkers

Serum Troponin I (cTN1), did not show any difference between groups (I/R versus sham-operated) (Table 1).

### 3.4. Biochemical Parameters (TNF-Alpha, MDA, and MPO)

**Figure 1:**
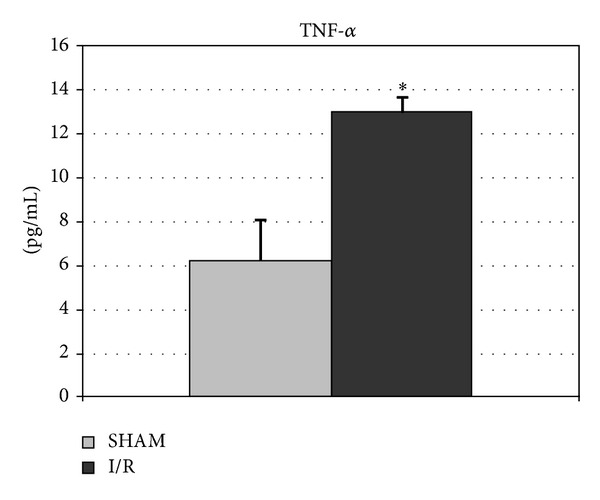
Serum levels of TNF-*α* in animals submitted to ischemia/reperfusion (I/R). Sham operated group (control, *n* = 15) has been compared with I/R group (*n* = 17): *P < 0.05. These are the mean results of 32 different experiments ± S.E.M.

The serum concentrations of TNF-alpha, an index of Kupffer cell activation, increased after ischemia/reperfusion as reported in Figure 1. No difference in MDA formation, as products of lipid peroxidation, was observed in liver and lung (Table 2). The same trend was found in kidney medulla and heart samples as compared with sham group (Heart: 0.113 ± 0.005 versus 0.122 ± 0.009, resp.; Kidney medulla 0.492 ± 0.098 versus 0.528 ± 0.129, resp.). The MDA levels were significantly higher in ischemic group compared with sham animals only in kidney cortex (0.205 ± 0.011 versus 0.142 ± 0.022 nmoli/mg/prot, *P* < 0.05).

Tissue MPO activity, an indirect evidence of neutrophil infiltration, was measured in liver and lung; mean MPO activity was increased in liver and lung comparing the ischemic group with sham animals (Table 2). No changes in the MPO levels were found in kidney and heart tissue during hepatic I/R injury, as compared with sham group (data not shown).

### 3.5. Gelatinolytic Activity

**Figure 2:**
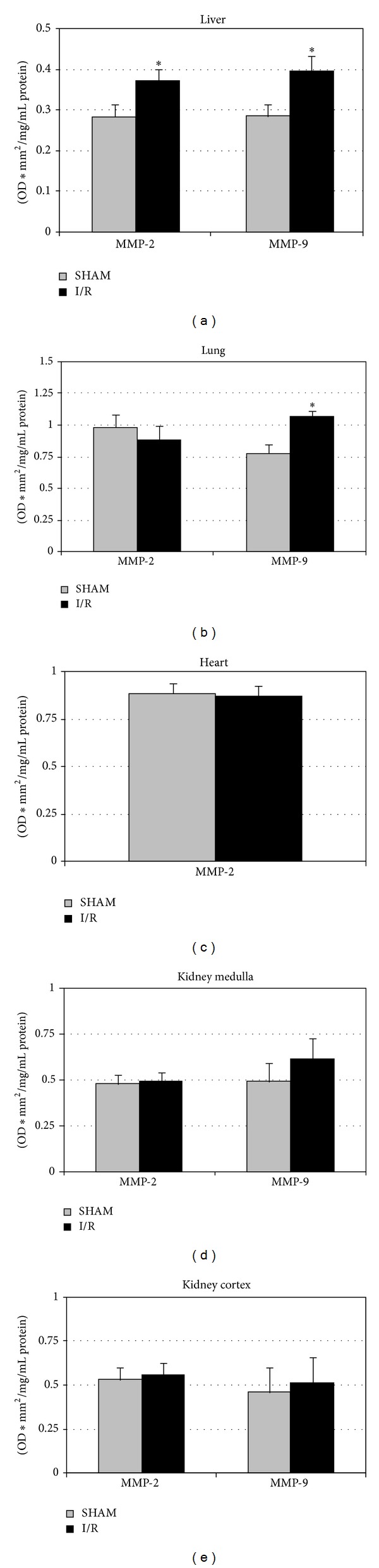
Bar graphs of MMP-2 and MMP-9 activity in ischemic liver lobe (a), lung (b), heart (c), kidney cortex (d), and medulla (e). Sham-operated group (control, *n* = 15) has been compared with I/R group (*n* = 17): *P < 0.05. Data are shown as mean values ± SEM.

The activity of gelatinase-A (MMP-2) and gelatinase-B (MMP-9) was evaluated to investigate the extent of hepatic, renal, lung, and heart MMPs activity, potentially inducing interstitial degradation (Figure 2). Both I/R and sham-operated groups showed detectable MMP-2 and MMP-9 activities with the exception of the heart where MMP-9 was not detectable.

In the liver, I/R was associated with a significant increase of gelatinase activity in the ischemic lobe (Figure 2). Interestingly, acute hepatic I/R was associated with lung MMP-9 activation, while in kidney (cortex and the medulla) and in heart no significant difference was observed between groups (I/R versus sham). Only a no significant increase in MMP-9 was observed in kidney medulla (Figure 2).

### 3.6. Liver Histology

**Figure 3:**
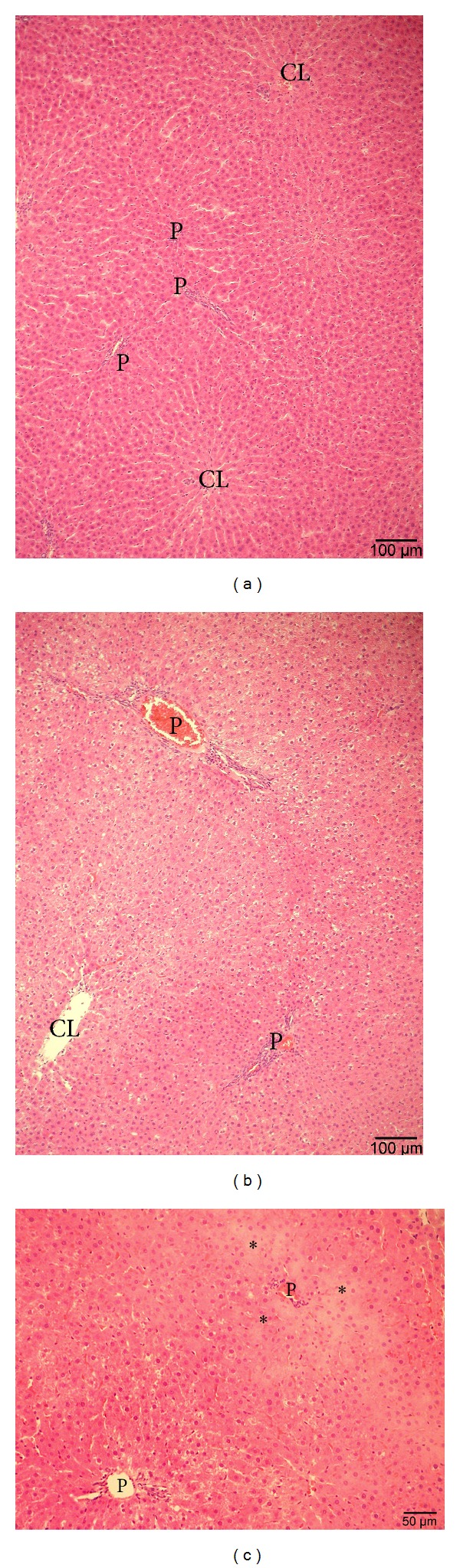
Representative light micrographs of the left liver lobe of sham-operated rats (a) and of the left lobe of rats submitted to ischemia/reperfusion (I/R) (b and c). Hematoxylin and eosin staining. The sham animal shows normal hepatocyte and sinusoid morphology (a). In animals submitted to I/R the lower magnification picture (b) shows decreased eosinophilia of hepatocytes, hepatocyte vacuolation, disarrangement of hepatocyte cords, and altered sinusoidal dilatation (b). An area of extensive hepatocyte necrosis and plate disintegration (black stars) is shown under higher magnification in (c), example of grade 3. P: portal vein; CL: centrolobular vein.

A semiquantitative evaluation of liver lesions showed a statistically significant difference in the extent of liver damage when comparing sham-operated rats and animals subjected to I/R (Score 0–3: 0.6 ± 0.1 versus 2.4 ± 0.2, resp.). In the ischemic lobe of the animals submitted to I/R several lobules showed a detectable damage. In particular, hepatocyte necrosis and sinusoidal disarrangement (Figures 3(b)-3(c), Grade 3) when compared with sham-operated animals (Figure 3(a)).

### 3.7. Kidney Histology

**Figure 4:**
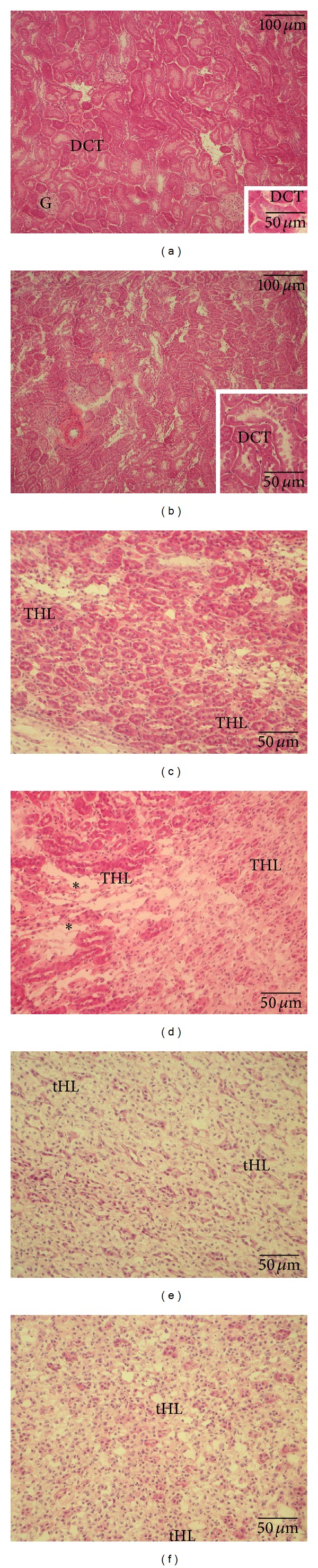
Representative light micrographs of kidney samples obtained from sham-operated rats (a, b, and c) and from rats submitted to hepatic ischemia/reperfusion (I/R) (d, e, and f). Cortex (a, b), outer medulla (c, d), and inner medulla (e; f) are illustrated. Hematoxylin and eosin staining. With respect to the normal morphology of the cortex of sham animals (a), the cortex of animals submitted to hepatic I/R (b) shows dilated interstitium and injury to a few tubules. The insets in (a) show a normal distal convolute tubule (DCT) and in (b) patchy areas of dilatation. With respect to the normal morphology of the outer medulla of sham animals (c), the outer medulla of animals submitted to hepatic I/R shows extended areas of interstitial fluid (asterisk) apparently displacing thick limbs of Henle's loop (THL). Respect to the inner medulla of sham animals (e), the inner medulla of animals submitted to I/R (f), shows slightly dilated thin limbs of Henle's loop (tHL) and increased cellularity in the stromal.

When compared with the cortex of sham-operated animals (Figure 4(a) and inset), the cortex of animals submitted to hepatic I/R showed dilated interstitium. Rats submitted to I/R showed damage to tubules with loss of brush border and presence of cellular debris (Figure 4(b) and inset). No significant damage to glomeruli was detected. With respect to the outer medulla of sham animals (Figure 4(c)), the outer medulla of rats submitted to hepatic I/R showed wider areas of interstitial fluid accumulation in the interstitium and disarrangement of thick limbs of Henle's loop (Figure 4(d)). In the inner medulla of animals submitted to I/R, a few thin limbs of Henle's loop appear dilated and with increased cellularity in the stromal (Figure 4(f)) with respect to sham animals (Figure 4(e)).

### 3.8. Lung Histology

**Figure 5:**
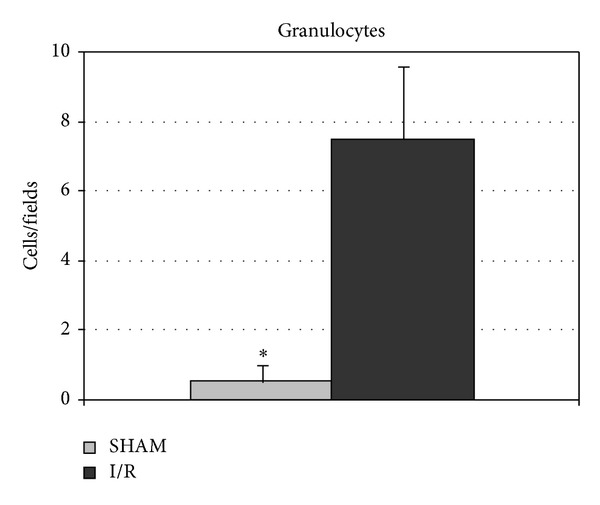
Changes of lung granulocytes in response to hepatic I/R injury. Lung samples were obtained from sham-operated rats, not submitted to hepatic I/R, and from rats whose hepatic lobes were submitted to 30 min of ischemia and hence reperfused for 60 min. The number of granulocytes was calculated per microscopic field. *P < 0.05. Data are shown as mean values ± SEM.

**Figure 6:**
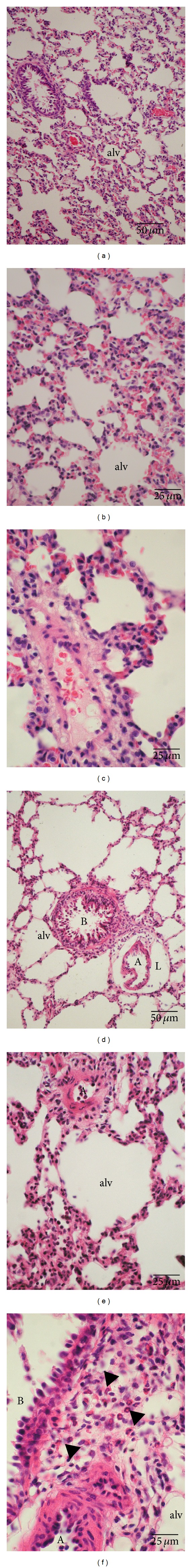
Representative sections of lung tissue from sham-operated animals (a, b, and c) and from animals submitted to hepatic ischemia/reperfusion (I/R) (d, e, and f). Hematoxylin and eosin staining. The sections from sham animals show the typical morphology of bronchi (B), blood vessels, and alveoli (alv) with associated capillaries; alveolar capillaries are recognizable in high magnification (c), by erythrocytes in the lumen. In the lung tissues of animals submitted to hepatic I/R alveoli appear to be dilated. (d, e) Dilated lymph vessels (L) surrounding arteries (A) and an abundant number of inflammatory cells (arrowheads) in the lumen and stroma of blood vessels (f).

A significantly higher number of granulocytes were found in the wall and lumen of alveoli of rats submitted to I/R as compared with sham rats (Figure 5). In contrast with the lungs of sham-operated animals (Figures 6(a), 6(b), and 6(c)), in the lungs of animals submitted to hepatic I/R alveoli appear to be dilated (Figures 6(d) and 6(e)), rare erythrocytes in alveolar capillaries (Figure 6(e)), dilated lymphatics (Figure 6(d)), and abundant granulocytes and mononuclear cells in the lumen and stroma of arteries (Figures 6(e) and 6(f)) and in the lumen of bronchi (not shown).

## 4. Discussion

This study shows that moderate acute hepatic ischemia/reperfusion (I/R) injury increases MMPs activity not only in the ischemic liver region but also in the lung, associated with histological damage in liver, lung, and kidney. The concomitant increase in serum TNF-alpha suggests a potential role for this cytokine in the development of multiorgan dysfunction arising from isolated hepatic I/R injury. A moderate hepatic I/R injury is able to increase MMPs activity not only in the ischemic region, as previously reported [29, 30], but also in the nonischemic lobe associated with several histological signs of interstitial and cellular damage [18]. This event is probably TNF-alpha-mediated, fully supporting the hypothesis that a direct connection exists between the events taking place in both the damaged lobe and the nonischemic liver.

### 4.1. The Involvement of Lung in the Hepatic I/R Injury

The results of the present work suggest that a sizeable release of hepatic enzymes in the blood stream following acute liver I/R injury is associated with increased MMP activity, in particular MMP-9 also in distant organs, such as the lung. Multiorgan failure (MOF) is the simultaneous dysfunction of several organs, and it represents one of the most intriguing clinical problems arising in patients admitted to the Intensive Care Unit [31]. A central mechanism leading to MOF seems to be I/R injury [32]. Oxygen-derived free radicals, cytokines, and activated neutrophils have been found to be involved in the I/R liver damage [33] triggering the systemic inflammatory response that contributes to distant organ injury [34]. Although the involved mechanism is still unclear, the observed increase in MMP-9 activity appears to be connected with the high serum levels of TNF-alpha representing the connection between the hepatic I/R damage and “at-a-distance” lung alterations.

MMPs are not expressed during normal conditions but their expression and activity increase during inflammation [35]. MMP-9 is one of the families of MMPs, which degrades ECM, and it is induced by many inflammatory factors, including IL-1beta, IL-8, and TNF-alpha. MMP-9 is stored in the tertiary granules of polymorphonuclear leukocytes which are key effectors in acute inflammatory diseases. In addition MMP-9 can actively assist MPO activation, an index of neutrophil infiltration [29]. Once an inflammatory response is initiated, neutrophils are the first cells to be recruited to the sites of injury or [7] infection [36]. After hepatic I/R injury liver MPO activity increased in the ischemic tissue and a few neutrophils were occasionally seen in edematous portal spaces, and/or forming small granulomas around necrotic cells in ischemic lobes. Interestingly, a similar increase of MPO levels was also observed in the lung tissue, associated with a high number of granulocytes as compared with the control group.

Previous data also show increased neutrophil infiltration in the liver and in other organs such as the lung after hepatic I/R, suggesting that neutrophils contribute to MOF induced by hepatic I/R [7]. In the present study, I/R injury was not associated with an oxidative damage in the liver and in the lung despite early signs of tissue damage. No significant difference was observed in MDA levels, a lipid peroxidation product, confirming our previous work in which liver MMP activation was shown to be an MDA-independent event [18]. A more prolonged reperfusion time is required to obtain a significant increase in MDA levels after hepatic I/R damage [37].

### 4.2. Kidney Involvement in the Hepatic I/R Injury

No increase in MMPs and MPO was observed in the kidney. Previously, Miranda et al. [38] demonstrated that 60 min hepatic ischemia associated with 2 or 6 hours reperfusion induced an increase in MPO and MDA in distant organ such as the lung and the kidney. In the present work we confirm the increase in pulmonary MPO and the absence of these alterations in other organs such as the kidney. A possible explanation is strictly connected with the short duration of both ischemia (30 min) and reperfusion (60 min) period in our experimental model, as well as with the much lower transaminase levels, 3-times lower than those observed by Miranda et al. [38]. No changes in MDA formation was found in distant organs such as the kidney, thus confirming the limited damage induced in our experimental setting, as supported by previous reports showing that the hepatic MDA levels after 60 min ischemia followed by 60 min reperfusion were comparable to those observed in sham animals [37]. We did only find increased MDA levels in the kidney cortex. Further studies will be performed to explain this finding. The histological analysis reveals some alterations such as focal patchy areas of dilatation and much higher fluid accumulation in rats submitted to I/R versus control group.

### 4.3. Heart Involvement in the Hepatic I/R Injury

No changes in heart MMPs, MDA, and MPO were observed, probably because cardiac dysfunction has been reported to follow only major liver surgical procedures when the liver is subjected to an important decrease in blood flow or after transplantation [39]. Meyer et al. [32] demonstrated that hepatic I/R induced the upregulation of ICAM, one of the adhesion molecules, mediated by TNF-alpha in distant organs such as the heart and the kidney. They evaluated the damage after 5-hour reperfusion and probably this is the reason why we did not find any cardiac alteration after 1-hour reperfusion; our hypothesis is supported by the strict correlation that exists between lipid peroxidation formation and ischemia time in the rat liver: only after 2 hours of reperfusion a marked MDA was shown [37].

### 4.4. Hepatic I/R Injury and MOF

Multiple organ dysfunction syndrome/failure is an important cause of death in the surgical intensive care unit. As a syndrome, MODS is defined as altered organ function in the setting of sepsis, septic shock, or systemic inflammatory response syndrome. Our data show that also after hepatic I/R, biochemical and histological changes do occur in distant organs and these events are traceable very early during reperfusion after a brief transient ischemia. A histopathology examination showed that hepatic, pulmonary, and renal tissue were more injured, albeit slightly, in the I/R rats than in sham animal. In particular increased MMP-9 activity was associated with early lung injury. Recently, some studies have shown that significant increases in active MMP-9 are associated with a multiple organ dysfunction in an infection model [40]. Our results suggest that after hepatic I/R an increased MMP-9 activation in distant organ can occur representing (a) a step forward the comprehension of the mechanisms involved in MOF and (b) an innovative target for limiting the MOF progression.

Interestingly, Rahman et al. recently demonstrated a novel role of MMPs in regulating infiltration of neutrophils by controlling platelets secretion of CD40L, a factor involved in the septic lung injury. Their results suggested that targeting MMPs may be a useful strategy for limiting lung injury [41]. Experiments are in progress in our laboratory for increasing both ischemia and reperfusion period.

Interestingly, the present work highlights that already after a relatively short I/R period an acute distant organ damage occurs and that the lung is the first organ involved, suggesting that the increase in lung MMP-9 activity may represent a key and early event involved in the pathogenesis of hepatopulmonary syndrome, whereas kidney injury may occur later and cardiac alterations may be observed only after a period of reperfusion longer than 1 hour [32].

The likelihood that an ischemic and reperfused organ can directly induce a remote organ failure is of a significant clinical importance: these effects must be taken into account when treating patients after liver transplantation and these findings may have important practical applications in the clinical management of liver transplantation, as well as in the procedures involving no flow-reflow conditions.

## Figures and Tables

**Table 1:** Serum levels of AST, ALT, LDH, creatinine, urea, and Troponin I in animals submitted to ischemia/reperfusion (I/R). Sham-operated group (control) has been compared with I/R group: *P < 0.05. These are the mean results of 32 different experiments ± S.E.M.

		SHAM (*n* = 15)	I/R (*n* = 17)
AST	mU/mL	118.92 ± 9.31	670.83 ± 180.33*
ALT	mU/mL	41.83 ± 3.48	609.17 ± 191.16*
LDH	mU/mL	2169.4 ± 486.2	8022.7 ± 2197.8*
Creatinine	mg/dL	0.67 ± 0.05	0.74 ± 0.04
Urea	mg/dL	48.33 ± 1.94	48.17 ± 1.7
Troponin I	ng/mL	0.02 ± 0.01	0.02 ± 0.01

**Table 2:** Liver and lung levels of MDA and MPO in animals submitted to ischemia/reperfusion (I/R). Sham-operated group (control) has been compared with I/R group: *P < 0.05. These are the mean results of 32 different experiments ± S.E.M.

		SHAM (*n* = 15)	I/R (*n* = 17)
MDA			
Liver	nmoli/mg/prot	0.292 ± 0.072	0.293 ± 0.062
Lung	nmoli/mg/prot	0.206 ± 0.022	0.210 ± 0.044

MPO			
Liver	nmoli/min/mL	1.7 ± 0.07	2.0 ± 0.06*
Lung	nmoli/min/mL	1.1 ± 0.01	1.2 ± 0.01*

## Abbreviations

MMP: Matrix metalloproteinases
I/R: Ischemia-reperfusion
MDA: Malondialdehyde
MPO: Myeloperoxidase.

## References

[1] Grace PA (1999). *Ischemia-Reperfusion Injury*.

[2] Carden DL, Granger DN (2000). Pathophysiology of ischaemia-reperfusion injury. *Journal of Pathology*.

[3] Oltean M, Mera S, Olofsson R (2006). Transplantation of preconditioned intestinal grafts is associated with lower inflammatory activation and remote organ injury in rats. *Transplantation Proceedings*.

[4] Behrends M, Hirose R, Park YH (2008). Remote renal injury following partial hepatic ischemia/reperfusion injury in rats. *Journal of Gastrointestinal Surgery*.

[5] Nielsen VG, Tan S, Baird MS, Samuelson PN, McCammon AT, Parks DA (1997). Xanthine oxidase mediates myocardial injury after hepatoenteric ischemia-reperfusion. *Critical Care Medicine*.

[6] Peralta C, Perales JC, Bartrons R (2002). The combination of ischemic preconditioning and liver Bcl-2 overexpression is a suitable strategy to prevent liver and lung damage after hepatic ischemia-reperfusion. *American Journal of Pathology*.

[7] Jiang H, Meng F, Li W, Tong L, Qiao H, Sun X (2007). Splenectomy ameliorates acute multiple organ damage induced by liver warm ischemia reperfusion in rats. *Surgery*.

[8] Colletti LM, Kunkel SL, Walz A (1995). Chemokine expression during hepatic ischemia/reperfusion-induced lung injury in the rat. The role of epithelial neutrophil activating protein. *Journal of Clinical Investigation*.

[9] Knittel T, Mehde M, Kobold D, Saile B, Dinter C, Ramadori G (1999). Expression patterns of matrix metalloproteinases and their inhibitors in parenchymal and non-parenchymal cells of rat liver: regulation by TNF-*α* and TGF-*β*1. *Journal of Hepatology*.

[10] Mignatti P, Rifkin DB (1999). Nonenzymatic interactions between proteinases and the cell surface: novel roles in normal and malignant cell physiology. *Advances in Cancer Research*.

[11] Moore C, Shen X-D, Gao F, Busuttil RW, Coito AJ (2007). Fibronectin-*α*4*β*1 integrin interactions regulate metalloproteinase-9 expression in steatotic liver ischemia and reperfusion injury. *American Journal of Pathology*.

[12] Kuyvenhoven JP, Ringers J, Verspaget HW, Lamers CBHW, Van Hoek B (2003). Serum matrix metalloproteinase MMP-2 and MMP-9 in the late phase of ischemia and reperfusion injury in human orthotopic liver transplantation. *Transplantation Proceedings*.

[13] Kuyvenhoven JP, Verspaget HW, Gao Q (2004). Assessment of serum-matrix metalloproteinases MMP-2 and MMP-9 after human liver transplantation: increased serum MMP-9 level in acute rejection. *Transplantation*.

[14] Leroy V, Monier F, Bottari S (2004). Circulating matrix metalloproteinases 1, 2, 9 and their inhibitors TIMP-1 and TIMP-2 as serum markers of liver fibrosis in patients with chronic hepatitis C: comparison with PIIINP and hyaluronic acid. *American Journal of Gastroenterology*.

[15] Kuroda T, Yoshida Y, Kamiie J (2004). Expression of MMP-9 in mesangial cells and its changes in anti-GBM glomerulonephritis in WKY rats. *Clinical and Experimental Nephrology*.

[16] Hochhauser E, Ben-Ari Z, Pappo O, Chepurko Y, Vidne BA (2005). TPEN attenuates hepatic apoptotic ischemia/reperfusion injury and remote early cardiac dysfunction. *Apoptosis*.

[17] Imberti R, Vairetti M, Gualea MR (1998). The effects of thyroid hormone modulation on rat liver injury associated with ischemia-reperfusion and cold storage. *Anesthesia and Analgesia*.

[18] Palladini G, Ferrigno A, Rizzo V (2012). Lobe-specific heterogeneity and matrix metalloproteinase activation after ischemia/reperfusion injury in rat livers. *Toxicologic Pathology*.

[19] Grisham MB, Anzueto Hernandez L, Granger DN (1986). Xanthine oxidase and neutrophil infiltration in intestinal ischemia. *American Journal of Physiology: Gastrointestinal and Liver Physiology*.

[20] Kossakowska AE, Edwards DR, Lee SS (1998). Altered balance between matrix metalloproteinases and their inhibitors in experimental biliary fibrosis. *American Journal of Pathology*.

[21] Lowry OH, Rosebrough NJ, Farr AL, Randall RJ (1951). Protein measurement with the Folin phenol reagent. *The Journal of Biological Chemistry*.

[22] Camp TM, Smiley LM, Hayden MR, Tyagi SC (2003). Mechanism of matrix accumulation and glomerulosclerosis in spontaneously hypertensive rats. *Journal of Hypertension*.

[23] Coker ML, Thomas CV, Clair MJ (1998). Myocardial matrix metalloproteinase activity and abundance with congestive heart failure. *American Journal of Physiology. Heart and Circulatory Physiology*.

[24] Kleiner DE, Stetler-Stevenson WG (1994). Quantitative zymography: detection of picogram quantities of gelatinases. *Analytical Biochemistry*.

[25] Tyagi SC, Campbell SE, Reddy HK, Tjahja E, Voelker DJ (1996). Matrix metalloproteinase activity expression in infarcted, noninfarcted and dilated cardiomyopathic human hearts. *Molecular and Cellular Biochemistry*.

[26] Tozzi R, Palladini G, Fallarini S (2007). Matrix metalloprotease activity is enhanced in the compensated but not in the decompensated phase of pressure overload hypertrophy. *American Journal of Hypertension*.

[27] Serafín A, Roselló-Catafau J, Prats N, Gelpí E, Rodés J, Peralta C (2004). Ischemic preconditioning affects interleukin release in fatty livers of rats undergoing ischemia/reperfusion. *Hepatology*.

[28] Cozzi E, Hazarika S, Stallings HW (2006). Ultrafine particulate matter exposure augments ischemia-reperfusion injury in mice. *American Journal of Physiology. Heart and Circulatory Physiology*.

[29] Hamada T, Fondevila C, Busuttil RW, Coito AJ (2008). Metalloproteinase-9 deficiency protects against hepatic ischemia/reperfusion injury. *Hepatology*.

[30] Hamada T, Duarte S, Tsuchihashi S, Busuttil RW, Coito AJ (2009). Inducible nitric oxide synthase deficiency impairs matrix metalloproteinase-9 activity and disrupts leukocyte migration in hepatic ischemia/reperfusion injury. *American Journal of Pathology*.

[31] Baue AE, Durham R, Faist E (1998). Systemic inflammatory response syndrome (SIRS), multiple organ dysfunction syndrome (MODS), multiple organ failure (MOF): Are we winning the battle?. *Shock*.

[32] Meyer K, Brown MF, Zibari G (1998). ICAM-1 upregulation in distant tissues after hepatic ischemia/reperfusion: a clue to the mechanism of multiple organ failure. *Journal of Pediatric Surgery*.

[33] Chen C-F, Wang D, Hwang CP (2001). The protective effect of niacinamide on ischemia-reperfusion-induced liver injury. *Journal of Biomedical Science*.

[34] Bhatia M, Moochhala S (2004). Role of inflammatory mediators in the pathophysiology of acute respiratory distress syndrome. *Journal of Pathology*.

[35] Nagase H, Visse R, Murphy G (2006). Structure and function of matrix metalloproteinases and TIMPs. *Cardiovascular Research*.

[36] Smith JA (1994). Neutrophils, host defense, and inflammation: a double-edged sword. *Journal of Leukocyte Biology*.

[37] Fukai M, Hayashi T, Yokota R (2005). Lipid peroxidation during ischemia depends on ischemia time in warm ischemia and reperfusion of rat liver. *Free Radical Biology and Medicine*.

[38] Miranda LEC, Capellini VK, Reis GS, Celotto AC, Carlotti CG, Evora PRB (2010). Effects of partial liver ischemia followed by global liver reperfusion on the remote tissue expression of nitric oxide synthase: lungs and kidneys. *Transplantation Proceedings*.

[39] Hochhauser E, Alterman I, Weinbroum A (2001). Effects of vasoactive substances released from ischemic reperfused liver on the isolated rat heart. *Experimental and Clinical Cardiology*.

[40] Teng L, Yu M, Li J-M (2012). Matrix metalloproteinase-9 as new biomarkers of severity in multiple organ dysfunction syndrome caused by trauma and infection. *Molecular and Cellular Biochemistry*.

[41] Rahman M, Roller J, Zhang S (2012). Metalloproteinases regulate CD40L shedding from platelets and pulmonary recruitment of neutrophils in abdominal sepsis. *Inflammation Research*.

